# Supplementary material for: Denoising diffusion-based MRI to CT image translation enables automated spinal segmentation
Source: Eur Radiol Exp. 2023 Nov 14;7:70. doi: 10.1186/s41747-023-00385-2 (PMC10643734; doi:10.1186/s41747-023-00385-2)
Supplement: Supplementary file 1 — Additional file 1. [file 41747_2023_385_MOESM1_ESM.docx]

## Denoising diffusion-based MRI to CT image translation enables automated spinal segmentation

## ELECTRONIC SUPPLEMENTARY MATERIAL

### Paired Image-2-Image

Generative adversarial networks were once the most popular image generation networks until denoising diffusion emerged. A GAN consists of a generator and a discriminator. The generator creates a new image, while the discriminator tries to distinguish between real and synthetic images. This approach leads to sharper images compared to earlier regressing networks like autoencoders, which only minimized a loss function between the generated and predicted image.

Paired image-to-image translation involves having data pairs representing views in distribution A and B. Pix2Pix [1] utilizes image pairs for a direct absolute difference loss, and to prevent blurry outputs, it adds a GAN-Loss to the optimization. However, acquiring paired data can be challenging, and despite Pix2Pix's relatively good performance for its age, there are few fundamentally new models. In GANs, balancing the predictive power of the generator and discriminator is often difficult. An imbalance can cause training to fail, and some GANs may suffer from mode collapse, where only a single image is generated repeatedly. A newer method called denoising diffusion overcomes this issue. For image-two-image translation, we condition our DDIM [2] by concatenating the image pair to the noised input [3], enabling us to learn paired image-to-image translation.

### Unpaired Image-2-Image

Producing unpaired image-to-image datasets is easier but comes with higher unreliability. Most variants use a Cycle-Consistency-Loss [4] to ensure related output images. This loss involves transferring an image to another domain and back, measuring the difference between the original and recreated images. However, this approach falls short when dealing with very imbalanced datasets, as the model may attempt to match the distributions of both domains, even if they are different. Consequently, issues like changing sizes, forgotten elements, or hallucinations may occur. We tested on a model called SynDiff [5], which is similar to CycleGAN. SynDiff includes a CycleGAN that generates image pairs for a DDPM [6]. The DDPM operates in image mode with a fixed step size of 4. On the other hand, CUT [7] stands out as it does not utilize Cycle-Consistency-Loss, but instead, it employs a contrastive loss. In certain layers, there is an enforcement that patches in the same region should be similar across different layers, whereas patches between different regions should be dissimilar.

### Denoising Diffusion

Denoising diffusion is a generative deep learning model. The model is forced to predict a Gaussian noise in an image where the noise has varying strengths. Gaussian noise is purely random, and the model has no choice but to learn how images of the dataset look like. Giving a random noise without an image causes the model to introduce image features into the noise. The noise strengths are defined in t timesteps. Where 0 is the image without noise; in time step t, the image is fully replaced by noise. We always used t=1000 for all experiments. From one step i-1 to i a Gaussian noise of $q\left( x_{i} | x_{i-1} \right)=\mathcal{N}\left( x_{i},\sqrt{1-\beta_{i}}x_{i-1},\beta_{i}I \right)$ is added where $\beta$ controls the strength of the noise. Starting at 0 for the noise-free image (i=0) and 1 for the completely noised image (i = t). We use a quadratic cosine curve for beta as in Nichol et al [8]. We optimize the model during training to predict the input noise $\epsilon$ or image $x_{0}.$As loss, we use the absolute difference loss. We can compute for any timestep a noised image with the “forward formula” $x_{i}=\sqrt{\bar{\alpha}_{i}}x_{0}+\sqrt{1-\bar{\alpha}_{i}}\varepsilon$ where $\varepsilon$ is a random normal distribution and$\alpha_{i}=1-\beta_{i};$ $\bar{\alpha}_{i}=\prod_{j=0}^{i} \alpha_{j}$. During inference, we iterate over the time steps. The model predicts either the noise $\hat{\epsilon}$ or the final image $\hat{x}_{0}$. We can compute the other by putting $x_{i}$ and $\hat{\epsilon}$ or $\hat{x}_{0}$ into the forward formula and solve for the missing value, like in the case of noise prediction: $\hat{x}_{0}=\frac{1}{\sqrt{\bar{\alpha}_{i}}}\left( x_{i} - \sqrt{1-\bar{\alpha}_{i}}\hat{\epsilon} \right)$. With $\hat{x}_{0}$ and $x_{i}$ we can compute the next $x_{j}$ for the time step j. The denoising diffusion probabilistic model (DDPM) [6] iterates over every step from t to 0. The predicted image $\hat{x}_{0}$ is mixed with $x_{i}$, and a new noise with an updated variance $\sigma$ must be applied. $x_{i-1}=\frac{1}{1-\bar{\alpha}_{i}}\left( \sqrt{\alpha_{i}}\left( 1-\bar{\alpha}_{i-1} \right)x_{i} +\sqrt{\bar{\alpha}_{i-1}}\beta_{i}\hat{x}_{0} \right) +\sigma\varepsilon$ where $\sigma= \sqrt{\beta_{i} (1-\bar{\alpha}_{i-1}) / (1-\bar{\alpha}_{i})}$. DDIM [2] reduces inference time by skipping an arbitrary amount of time steps. It also introduces a parameter $\eta$. For $\eta=1$, we use a random noise $\varepsilon$ in each step, while for $\eta=0$, we reuse the predicted noise $\hat{\epsilon}$. The forward step from step i to j (i>j) is: $\sqrt{\bar{\alpha}_{j}}\hat{x}_{0}+c_{1}\varepsilon+c_{2}\hat{\epsilon}$where $c_{1}=\eta\sqrt{\left( \frac{1-\bar{\alpha}_{j}}{1-\bar{\alpha}_{i}} \right)\left( 1-\frac{\bar{\alpha}_{i}}{\bar{\alpha}_{j}} \right)}$ and $c_{2}= \sqrt{(1-\bar{\alpha}_{j}) - {c_{1}}^{2}}$. We clamp $\hat{x}_{0}$ to -1 and 1 because we know we are limited to this range.

### Hyperparameters - SA-UNet

We copied the model parameters from <https://github.com/lucidrains/denoising-diffusion-pytorch>. We reimplemented the DDIM method to get feature parity to DDPM implementation. The SA-UNet is an often-used architecture for denoising diffusion. [8, 9] It uses self-attention blocks and repeatedly feeds the timestep t as a cosine embedding into the residual blocks. We used a starting channel size of 64. For the details, please refer to our GitHub implementation.

The input is the noised target image and the image of the other domain. The output is the prediction of the noise or denoised image. For the generation, we start with a Gaussian noise and the source image. We use the cosine schedule by Nichol and Dhariwal [8]. We use 1000 sampling steps as a goal for training. For sampling, we use the denoising diffusion implicit formulation. [2, 6] We can reduce this number of steps to 20 without observing a noticeable drop in translation quality. We did not do any further hyperparameter searches for the 3D variants. For the 3D version, we saw that we had to change t to at least 25.

### Hyperparameters – Others

We needed a hyperparameter search for the reference implementation of ResNet and UNet, unlike the SA-UNet, which only required finding a suitable learning rate. For Pix2Pix, we found that a basic UNet works best from the reference implementation. We used five down and up blocks. The batch size was 64 and the dropout was 20 %. For CUT, the optimal hyperparameter was especially difficult. We found that a ResNet with only one down/up convolution works best. We used a batch size of 32 and 8 residual Blocks. We turned off the dropout. The contrastive loss was computed on the 0,4,8,12,16 layers of the reference implementation. [7] The SA-UNet had its contrastive loss computed after every down-convolution and the first up-convolution. The rest of the model is equal to the diffusion implementation. The parameters were fixed on the original T1 dataset. A change in data causes notable differences in performance for CUT and every dataset would have required its own hyperparameter tuning. We had to freeze the parameters for a fair comparison of the model and not to be influenced by our ability to find hyperparameters in an unstable environment.

We used the same discriminator as the reference implementations with three down convolution blocks. [1] The number of channels was the same as the generator network.

### Ablation DDIM Inference

Denoising diffusion was trained to predict a noise at 1000 different strengths. The original denoising diffusion probabilistic model (DDPM) [6] iterates through all noise strengths from the strongest to the weakest noise. DDIM [2] is a reformulation of DDPM and skips an arbitrary number of intermediate noise levels on the same trained network. This reduces the required iteration from 1000 to t. We used t=20 in this paper in 2D and t=25 for 3D. With the reformulated forward process DDIM we get better results than the slower DDPM (**T1**=27.18; **T2**=26.87; p<0.001). For 3D translation, we observe that the spine fully forms with 25 or more inference steps. The background and skull bone were not fully formed with t=25 and would require more timesteps. DDIM has an additional feature where instead of pulling a random noise in every step, we can use only a random noise in the initial step and the other is computed from the previous input and the model prediction. This makes the inference deterministic and can enable interpolation of the DDIM output. This behavior is handled by the parameter h. h=0 means fully deterministic and h=1 means that every step receives a fully random noise. A third inference parameter is classifier free-guidance w [10]. If w is not zero, we sample the model in each step twice. One receives the conditional MRI input, and the other receives a black image. The conditioned output is multiplied by w+1 and the unconditioned output is multiplied by -w. Both are added together. The idea is to push the output away from the general bias of the network towards the condition. All three parameters can be used on a DDPM trained network without requiring retraining and are tested on the 2D network exclusively.

We did an ablation on our DDIM. We only changed one parameter and kept the rest fixed on w=0, h=1 and t=20. We choose two numbers of timesteps for the ablation t=10, t=20, and t=50. The results lead to no conclusion if there is a better t in image quality. We could reduce the t even further than 20 without sacrificing quality. An h of zero has a small negative impact (**T1**=27.48; **T2**=26.81 p<0.001) in noise mode and has a positive impact in image mode (**T1**=27.95; **T2**=27.41; p<0.001). We suspect that the parameters t and h have a too minor impact and we cannot say in general what value must be set to get an optimal result. The classifier free-guidance has a small impact on the test data and we see no pattern if any w improves the image quality. We turned off the classifier free-guidance for our 3D models.

The inference hyperparameter of DDIM image mode did not impact the segmentation results, while there are noticeable differences for DDIM in noise mode. The DDIM with t=10 (T1=0.81, T2=0.75, MRSSegClg=0.77) had the best scores in image quality but was the lowest performing inference type in the Dice metric (t=10 vs. t=20 p=0.09). The parameter h impacted the Dice score in an inconsistent way. We see no correlation between small differences in the quality metrics and Dice scores.

### Computational Efficiency

We observed that the 3D diffusion converged on a single V40 / RTX 3090 after four days. Which is notably less than the 17.5 days reported by Bieder et al. [11]. The 2D diffusion converged after 2-3 days. Our denoising diffusion models were trained on small batch sizes (2 in 3D and 8 in 2D images) and relatively short compared to other studies. The reason is the single channel output and the image condition easing the diffusion training. For medical data, the image condition reduced the training time by at least half compared to without. On multi-channel images, we noticed a color drift in individual channels in the same magnitude as random Gaussian noise when we trained on batches with 8 batches instead of the recommended 2048. The color drift is moving during training but is mostly fixed during inference. This causes the images to be biased like all images are too red or bright. This effect accumulates, meaning reducing the number of steps reduces the impact of this bias.

**Table S. 1:** Image Quality for T1w and T2w to CT Translation for different quality measures and with DDIM inference ablation.

| **From T1w** | **L1↓** | **MSE ↓** | **PSNR↑** | **SSIM↑** | **VIFp ↑** |
| --- | --- | --- | --- | --- | --- |
| **CUT ResNN (unpaired)** | 0.0224 | 0.0050 | 23.50 | 0.835 | 0.295 |
| CUT SA-UNet (unpaired) | 0.0295 | 0.0083 | 21.76 | 0.819 | 0.269 |
| **Pix2Pix UNet** | 0.0143 | 0.0023 | 27.37 | 0.881 | 0.392 |
| Pix2Pix SA-UNet | 0.0135 | **\0.0020** | 27.82 | 0.883 | 0.394 |
| **SynDiff (unpaired)** | 0.0150 | 0.0024 | 27.01 | 0.865 | 0.373 |
| DDIM noise h=1, t=10, w=0 | 0.0135 | 0.0021 | 27.64 | 0.877 | 0.395 |
| DDIM noise h=0, t=20, w=0 | 0.0139 | 0.0022 | 27.48 | 0.875 | 0.388 |
| **DDIM noise h=1, t=20, w=0** | 0.0136 | 0.0021 | 27.60 | 0.879 | 0.396 |
| DDIM noise h=1, t=20, w=1 | 0.0140 | 0.0023 | 27.33 | 0.880 | 0.394 |
| DDIM noise h=1, t=20, w=2 | 0.0144 | 0.0024 | 27.02 | 0.878 | 0.388 |
| DDIM noise h=1, t=50, w=0 | 0.0139 | 0.0022 | 27.48 | 0.880 | 0.395 |
| DDPM | 0.0146 | 0.0023 | 27.18 | 0.873 | 0.381 |
| DDIM image h=1, t=10, w=0 | **\0.0130** | **\0.0020** | **\28.00** | 0.887 | 0.411 |
| DDIM image h=0, t=20, w=0 | **\0.0130** | **\0.0020** | 27.95 | **\0.889** | **\0.415** |
| **DDIM image h=1, t=20, w=0** | 0.0131 | **\0.0020** | 27.89 | 0.887 | 0.411 |
| DDIM image h=1, t=20, w=1 | 0.0134 | 0.0021 | 27.65 | 0.887 | 0.408 |
| DDIM image h=1, t=20, w=2 | 0.0145 | 0.0025 | 26.88 | 0.885 | 0.398 |
| DDIM image h=1, t=50, w=0 | 0.0136 | 0.0022 | 27.57 | 0.885 | 0.407 |

| **From T2w** | **L1↓** | **MSE ↓** | **PSNR↑** | **SSIM↑** | **VIFp ↑** |
| --- | --- | --- | --- | --- | --- |
| **CUT ResNN (unpaired)** | 0.0213 | 0.0046 | 23.72 | 0.848 | 0.312 |
| CUT SA-UNet (unpaired) | 0.0215 | 0.0046 | 23.75 | 0.850 | 0.311 |
| **Pix2Pix UNet** | 0.0142 | 0.0023 | 26.95 | 0.895 | 0.392 |
| Pix2Pix SA-UNet | 0.0142 | 0.0023 | 26.87 | 0.890 | 0.384 |
| SynDiff (unpaired) | 0.0140 | 0.0022 | 27.12 | 0.885 | 0.385 |
| DDIM noise h=1, t=10, w=0 | 0.0145 | 0.0026 | 26.49 | 0.891 | 0.387 |
| DDIM noise h=0, t=20, w=0 | 0.0142 | 0.0024 | 26.81 | 0.888 | 0.382 |
| **DDIM noise h=1, t=20, w=0** | 0.0139 | 0.0023 | 26.92 | 0.894 | 0.391 |
| DDIM noise h=1, t=20, w=1 | 0.0140 | 0.0023 | 26.81 | 0.894 | 0.386 |
| DDIM noise h=1, t=20, w=2 | 0.0144 | 0.0024 | 26.59 | 0.891 | 0.379 |
| DDIM noise h=1, t=50, w=0 | 0.0138 | 0.0022 | 26.99 | 0.894 | 0.389 |
| DDPM | 0.0141 | 0.0023 | 26.87 | 0.890 | 0.381 |
| DDIM image h=1, t=10, w=0 | 0.0131 | **\0.0020** | 27.39 | 0.898 | 0.401 |
| DDIM image h=0, t=20, w=0 | **\0.0130** | **\0.0020** | **\27.41** | **\0.900** | **\0.404** |
| **DDIM image h=1, t=20, w=0** | 0.0131 | 0.0021 | 27.36 | 0.898 | 0.401 |
| DDIM image h=1, t=20, w=1 | 0.0133 | 0.0021 | 27.27 | 0.897 | 0.398 |
| DDIM image h=1, t=20, w=2 | 0.0139 | 0.0023 | 26.89 | 0.894 | 0.387 |
| DDIM image h=1, t=50, w=0 | 0.0132 | 0.0021 | 27.31 | 0.898 | 0.401 |
|  |  |  |  |  |  |
| Pix2Pix 3D | 0.0188 | 0.0039 | 26,38 | 0.889 | 0.428 |
| DDIM 3D noise h=1, t=25 | 0.0194 | 0.0041 | 26,22 | 0.894 | 0.444 |
| DDIM 3D image h=1, t=25 | 0.0189 | 0.0040 | 26,22 | 0.892 | 0.434 |

Note. — Arrows indicate if smaller or bigger is better. As a visual aid, we marked the best values with **\**. We marked multiple values if they were below the rounding threshold. *CUT contrastive unpaired translation, DDIM denoising diffusion implicit model, DDPM denoising diffusion probabilistic model, MSE mean squared error, PSNR peak signal-to-noise ratio, SA-UNet self-attention U-network, SSIM structural similarity index metric, VIFp visual information fidelity*

**Table S. 2:** Average Dice score↑ per Volume and per Vertebra on the T1w, T2w, and the MRSSegClg. MRSSegClg (ours) is a split where we improved the segmentation to better align the segmentation with the actual bone structure. This table includes the ablation of DDIM inference.

|  | **per vol.** | **per vert.** | **per vol.** | **per vert.** | **per vol.** | **per vert.** | **per vol.** | **per vert.** |
| --- | --- | --- | --- | --- | --- | --- | --- | --- |
| **Dataset** | **T1w** | **T1w** | **T2w** | **T2w** | **MRSSegClg** | **MRSSegClg** | **MRSSegClg (our)** | **MRSSegClg (our)** |
| **CUT ResNN (unpaired)** | 0.30 | 0.28 | 0.49 | 0.46 | 0.54 | 0.49 | 0.54 | 0.50 |
| CUT SA-UNet (unpaired) | 0.09 | 0.08 | 0.26 | 0.23 | 0.02 | 0.01 | 0.03 | 0.02 |
| **Pix2Pix UNet** | 0.79 | 0.80 | 0.73 | 0.69 | 0.75 | 0.74 | 0.76 | 0.76 |
| Pix2Pix SA-UNet | **\0.82** | 0.82 | 0.75 | 0.72 | **\0.77** | **\0.76** | 0.77 | 0.77 |
| **SynDiff (unpaired)** | 0.80 | 0.81 | **\0.77** | **\0.74** | **\0.77** | **\0.76** | 0.77 | 0.76 |
| DDIM noise h=1, t=10, w=0 | 0.76 | 0.77 | 0.65 | 0.61 | 0.74 | 0.72 | 0.77 | 0.78 |
| DDIM noise h=0, t=20, w=0 | 0.78 | 0.78 | 0.70 | 0.67 | 0.75 | 0.73 | 0.77 | 0.77 |
| **DDIM noise h=1, t=20, w=0** | 0.78 | 0.77 | 0.72 | 0.69 | 0.75 | 0.73 | 0.77 | 0.78 |
| DDIM noise h=1, t=20, w=1 | 0.80 | 0.80 | 0.74 | 0.71 | 0.76 | 0.74 | 0.77 | 0.77 |
| DDIM noise h=1, t=20, w=2 | 0.80 | 0.81 | 0.74 | 0.71 | 0.76 | 0.74 | 0.76 | 0.76 |
| DDIM noise h=1, t=50, w=0 | 0.81 | 0.82 | 0.73 | 0.70 | 0.76 | 0.74 | 0.77 | 0.78 |
| DDIM image h=1, t=10, w=0 | 0.81 | 0.82 | 0.75 | 0.72 | **\0.77** | **\0.76** | **\0.78** | **\0.79** |
| DDIM image h=0, t=20, w=0 | **\0.82** | 0.82 | 0.75 | 0.72 | **\0.77** | **\0.76** | **\0.78** | 0.78 |
| **DDIM image h=1, t=20, w=0** | **\0.82** | **\0.83** | 0.75 | 0.72 | **\0.77** | **\0.76** | **\0.78** | 0.78 |
| DDIM image h=1, t=20, w=1 | **\0.82** | **\0.83** | 0.75 | 0.72 | **\0.77** | **\0.76** | **\0.78** | 0.78 |
| DDIM image h=1, t=20, w=2 | **\0.82** | **\0.83** | 0.75 | 0.72 | **\0.77** | 0.75 | 0.77 | 0.78 |
| DDIM image h=1, t=50, w=0 | 0.81 | 0.81 | 0.75 | 0.72 | **\0.77** | **\0.76** | **\0.78** | 0.78 |

Note. — We marked the best values with **\.** *CUT contrastive unpaired translation, DDIM denoising diffusion implicit model, DDPM denoising diffusion probabilistic model, MRSSegClg MRSpineSeg Challenge, SA-UNet self-attention U-network, vert. vertebra, vol. volume*

## References

1. Isola P, Zhu J-Y, Zhou T, Efros AA (2017) Image-to-image translation with conditional adversarial networks. In: Proc. IEEE Comput. Soc. Conf. Comput. Vis. Pattern Recognit (CVPR), 2017. pp 1125-1134. https://doi.org/10.1109/CVPR.2017.632

2. Song J, Meng C, Ermon S (2021) Denoising diffusion implicit Models. In: International Conference on Learning Representations (ICLR), 2021. p https://doi.org/10.48550/arXiv.2010.02502

3. Saharia C, Chan W, Chang H, et al (2022) Palette: Image-to-image diffusion models. In: ACM SIGGRAPH 2022 Conference Proceedings. pp 1-10. https://doi.org/10.1145/3528233.3530757

4. Zhu J-Y, Park T, Isola P, Efros AA (2017) Unpaired image-to-image translation using cycle-consistent adversarial networks. In: Proc. IEEE Int. Conf. Comput. Vis. pp 2223-2232. https://doi.org/10.1109/ICCV.2017.244

5. Özbey M, Dalmaz O, Dar SU, et al (2023) Unsupervised medical image translation with adversarial diffusion models. IEEE Trans Med Imaging. https://doi.org/10.1109/TMI.2023.3290149

6. Ho J, Jain A, Abbeel P (2020) Denoising diffusion probabilistic models. In: Larochelle H, Ranzato M, Hadsell R, et al (eds) Proceedings of the 34th International Conference on Neural Information Processing Systems (NeurIPS 2020). Curran Associates, Inc., pp 6840–6851, https://doi.org/10.48550/arXiv.2006.11239

7. Park T, Efros AA, Zhang R, Zhu J-Y (2020) Contrastive learning for unpaired image-to-image translation. In: Proceedings of the IEEE International Conference on Computer Vision (ECCV), 2020. Springer, pp 319-345. https://doi.org/10.1007/978-3-030-58545–7_19

8. Nichol AQ, Dhariwal P (2021) Improved denoising diffusion probabilistic models. In: Proceedings of the 38th International Conference on Machine Learning. PMLR, pp 8162-8171. https://doi.org/10.48550/arXiv.2102.09672

9. Dhariwal P, Nichol A (2021) Diffusion models beat gans on image synthesis. In: Adv. Neural Inf. Process. Syst. 34 (NeurIPS 2021). pp 8780-8794. https://doi.org/10.48550/arXiv.2105.05233

10. Ho J, Salimans T (2022) Classifier-free diffusion guidance. arXiv preprint arXiv:220712598

11. Bieder F, Wolleb J, Durrer A, et al (2023) Diffusion models for memory-efficient processing of 3D medical images. arXiv preprint arXiv:230315288 https://doi.org/10.48550/arXiv.2303.15288
